# Balancing consistency and flexibility: exploring trait and state optimism in rats using the cognitive judgement bias test

**DOI:** 10.3389/fvets.2026.1849399

**Published:** 2026-07-24

**Authors:** Louisa Bierbaum, Rebecca Anna Mainzer, Carolin Mundinger, Sylvia Kaiser, S. Helene Richter

**Affiliations:** 1 Department of Behavioural Biology, University of Münster, Münster, Germany; 2 Joint Institute for Individualisation in a Changing Environment (JICE), University of Münster and Bielefeld University, Bielefeld, Germany

**Keywords:** animal personality, cognitive judgement bias, optimism, rats, spatial task, state, trait, ultrasonic vocalizations

## Abstract

Optimistic or pessimistic interpretation of ambiguous information has been widely used in animal welfare science as an indicator of emotional states. Yet, growing evidence suggests that besides this state dimension, animals differ consistently in their optimism levels, suggesting a trait dimension of optimism, too. The boundary as well as a potential interplay between these two dimensions, however, remains poorly understood. Here, we aimed to disentangle consistent trait from transient state optimism using a spatial cognitive judgement bias test in laboratory rats. Specifically, we repeatedly tested individuals for their optimism levels and assessed the repeatability to infer trait optimism. Then, we exposed the rats to either negatively valenced 22 kHz or positively valenced 50 kHz USV playbacks to manipulate state optimism. Lastly, we conducted tests for anxiety-like behavior to assess whether the manipulation was accompanied by broader changes consistent with altered emotional state. Building on previous work, we found repeatable optimism levels over 3 weeks, supporting the existence of a trait dimension of optimism. Surprisingly, however, we did not detect any statistically significant effects of the USV playbacks on optimism levels and anxiety-like behavior, hindering the systematic investigation of a state dimension. Overall, our study provides further evidence that optimism has a consistent trait dimension, while highlighting the ongoing need for future research to successfully disentangle trait and state components of behavior.

## Introduction

The popular example of the half-filled glass of water and its interpretation as “half full” or “half empty” illustrates how people differ in the way they judge ambiguous information. Commonly, these different interpretations are referred to as more “optimistic” or more “pessimistic” judgments (terms hereafter used without quotation marks). Scientifically, optimism and pessimism thereby reflect an individual’s general tendency to expect either rewarding or aversive outcomes in ambiguous situations and can be conceptualized as two opposing ends of a spectrum (1). For simplicity, however, we will refer to an individual’s position on this spectrum only as its “optimism level” in the following. Adopting this concept from human psychology, optimistic and pessimistic decision-making has been used as a proxy in animal welfare science to assess emotional states of animals (2). More specifically, so-called “cognitive judgement bias tests” have been developed to analyze an animal’s response toward ambiguous stimuli. Thereby, animals interpreting ambiguous cues more optimistically are assumed to be in a more positive emotional state than animals interpreting ambiguous cues more pessimistically (3).

Typically, animal welfare studies make use of such cognitive judgement bias tests to investigate modulations of emotional state, for instance, by changing environmental enrichment [e.g., (4–6)], mimicking play behavior (7) or inflicting pain (8). Hereby, “state” refers to a transient and context-dependent condition, observable only at specific moments in time and influenced by situational factors (9–11). However, more recently, researchers have started to also investigate a potential “trait” dimension of optimism and pessimism. This interest builds on earlier work on individual differences in behavior that are consistent across time and/or contexts, commonly referred to as animal personality in the literature (12–15). Indeed, individuals of the same population have been found to differ consistently in their behavior, with some individuals being shyer, some bolder, some less and some more aggressive than others (13, 16, 17). Correspondingly, temporally consistent inter-individual differences in optimism levels have been reported for 3 days in bottlenose dolphins (18) and wild house mice (19) and for 25 days in dairy calves (20). In laboratory rodents, optimism levels were even found to be consistent across 7 weeks in mice (21) and for a time span of 10 weeks in rats (22).

Together, this evidence supports the assumption that cognitive judgement bias can comprise both a trait and a state dimension (23, 24). “State optimism” thereby captures optimism levels shown at particular moments in time and is potentially modulated by situational factors, while “trait optimism” describes an individual’s general tendency to make more optimistic or more pessimistic judgments, i.e., to behave consistently over time. Clearly distinguishing between state and trait optimism, however, remains difficult since the line between the two dimensions appears to be blurred. For instance, trait optimism may only stabilize with increasing age (22), which complicates the distinction between state-dependent fluctuation and not fully established trait optimism. Besides that, trait and state optimism are also likely to interact with each other, since a trait optimist may react differently to a negative or positive experience than a trait pessimist. Rygula and colleagues (25), for example, showed that rats characterized as trait pessimists exhibited greater levels of stress-induced anhedonia following restraint stress than trait optimists. At the same time, restraint stress led to a greater negative cognitive bias in all animals, regardless of underlying trait optimism or pessimism. However, hardly any further studies have systematically examined both trait and state optimism as well as possible interactions within a single study.

To disentangle potential trait and state dimensions of cognitive judgement bias, we here aimed to systematically investigate how both a modulation of state optimism via positive and negative experiences as well as the underlying trait optimism influence optimism levels in laboratory rats (*Rattus norvegicus* f. *domestica*). Therefore, we first repeatedly tested rats in the cognitive judgement bias test to assess their trait optimism. Next, we aimed at modulating their state optimism exposing them to positive and negative experiences. For that purpose, we chose to use different playbacks of ultrasonic vocalization (USVs) which have already been shown to modulate optimism levels in rats. More specifically, Saito and colleagues (26) subjected rats to 22 kHz or 50 kHz vocalizations along with a control group without USV playback and conducted a cognitive judgement bias test. They showed that rats exposed to 22 kHz USV playbacks were more pessimistic than rats subjected to 50 kHz playbacks and the control group. In contrast, rats which were presented with 50 kHz vocalizations were more optimistic than the control group.

Overall, appetitive 50 kHz vocalizations are widely thought to indicate positive emotional states (27–29) and are produced in rewarding contexts, including mating (30, 31), social play (32) and tickling, which imitates social play (33). They are emitted to communicate these positive states to conspecifics and to coordinate social behavior (34–37). ln line with this, playback of 50 kHz vocalizations can elicit approach behavior and increase exploration (38–41). In contrast, aversive 22 kHz vocalizations are assumed to reflect negative emotional states (27–29) and are typically emitted in threatening or stressful situations, such as encounters with a dominant and aggressive conspecific (42), during the presence of a predator (43) or when in pain (44). Against the background of social communication, 22 kHz vocalizations are considered alarm calls (45) and playback of these USVs can induce avoidance behavior, reduced locomotion and freezing (46, 47).

Building on this, we expected rats to differ consistently in optimism levels across repeated cognitive bias tests (i.e., trait optimism) and to respond more pessimistically following 22 kHz playback than following 50 kHz playback (i.e., state optimism). Moreover, we hypothesized trait optimism to interact with the two USV playback treatments, resulting in varying optimism levels. Lastly, we conducted a battery of standard behavioral tests under USV playback exposure to further study the effects of the different USV treatments on the animals’ state. Since playback of these vocalizations has been shown to influence anxiety-like behavior (48–50), we presumed rats subjected to 22 kHz playbacks to show more anxiety-like behavior than those exposed to a 50 kHz treatment.

## Materials and methods

### Animals and housing conditions

Twenty-four female Lister Hooded rats (LIS: Crl) were obtained from a professional breeder (Charles River Laboratories, Research Models and Services, Germany GmbH, Sulzfeld, Germany) at 4 weeks of age. Only female rats were used in the present study because our aim was to establish a proof of principle rather than to investigate sex differences. Moreover, although recent studies have increasingly included female rodents in cognitive judgement bias research [e.g., (21, 22)], female rodents have historically been underrepresented in this field (51). The rats arrived in four batches, with the first two batches consisting of four animals and the last two batches consisting of eight animals. Batches were tested successively without overlapping experimental phases. They were housed in groups of four per cage and identified based on their fur patterns. During the course of the experiment, two animals had to be sacrificed due to unrelated health problems and were excluded from the analysis. The cages (“Furat” model, ferplast S.p.A., Castelgamberto, Italy; 78 × 48 × 70 cm^3^) had wood shavings as bedding material (TierWohl Super, J. Rettenmaier and Söhne GmbH & Co KG, Rosenberg, Germany) and two additional levels to facilitate climbing and jumping. To enhance enrichment and provide shelter, the cages were equipped with a transparent red plastic tunnel (ZOONLAB GmbH Animal Husbandry Experts, Germany; 15 × 9 × 9.5 cm), a plastic house (ZOONLAB GmbH Animal Husbandry Experts, Germany; 20.5 × 15.7 × 11.5 cm), a cardboard tunnel (ZOONLAB GmbH Animal Husbandry Experts, Germany; 12.5 cm long, Ø 9 cm), and two plastic hammocks (Sputnik, SAVIC, Belgium; 29 × 26 × 19 cm) suspended from the cage top. Additionally, paper towels and four wooden cubes were provided. The housing environment was maintained at approximately 22 °C with a relative humidity of around 50%. A reversed 12:12 h light/dark cycle was implemented, with lights off at 9:15 a.m. Food (Altromin 1,324, Altromin GmbH, Germany) and water were available *ad libitum* until the start of the experimental phase. During the CJB training and testing phases, the rats were subjected to a mild food restriction, receiving food once daily after training or testing. At an age of 10 weeks, their current body weight was determined and used as a reference to keep them on 90–95% of that weight. This controlled food restriction aimed to enhance motivation for food-based tasks while also preventing overfeeding, which is known to improve laboratory rodents’ overall health (52, 53). Body weight was monitored daily, with a maximum break of 2 days, using a digital scale (PCE-BT 2000, PCE Deutschland GmbH, Meschede, Germany; capacity: 2,100 g, resolution: 0.01 g). The amount of food provided per cage was determined based on the weight of the lightest rat within each group.

### Experimental design

To determine trait optimism, the animals were tested twice in the cognitive judgement bias test within an initial characterization phase. Then, before entering the treatment phase, the animals were evenly distributed to the two different USV experiences, 22 kHz and 50 kHz playbacks, based on their optimism levels (see “Allocation of individuals to USV treatments” for further details). The different USV experiences thereby served as positive and negative experiences supposed to induce changes in state optimism [cf. (26)]. During the treatment phase, the animals were then exposed to the USV playbacks while undergoing another cognitive judgement bias test. Additionally, the rats participated in a battery of standard behavioral tests while being subjected to the respective USV experience.

**Figure 1:**
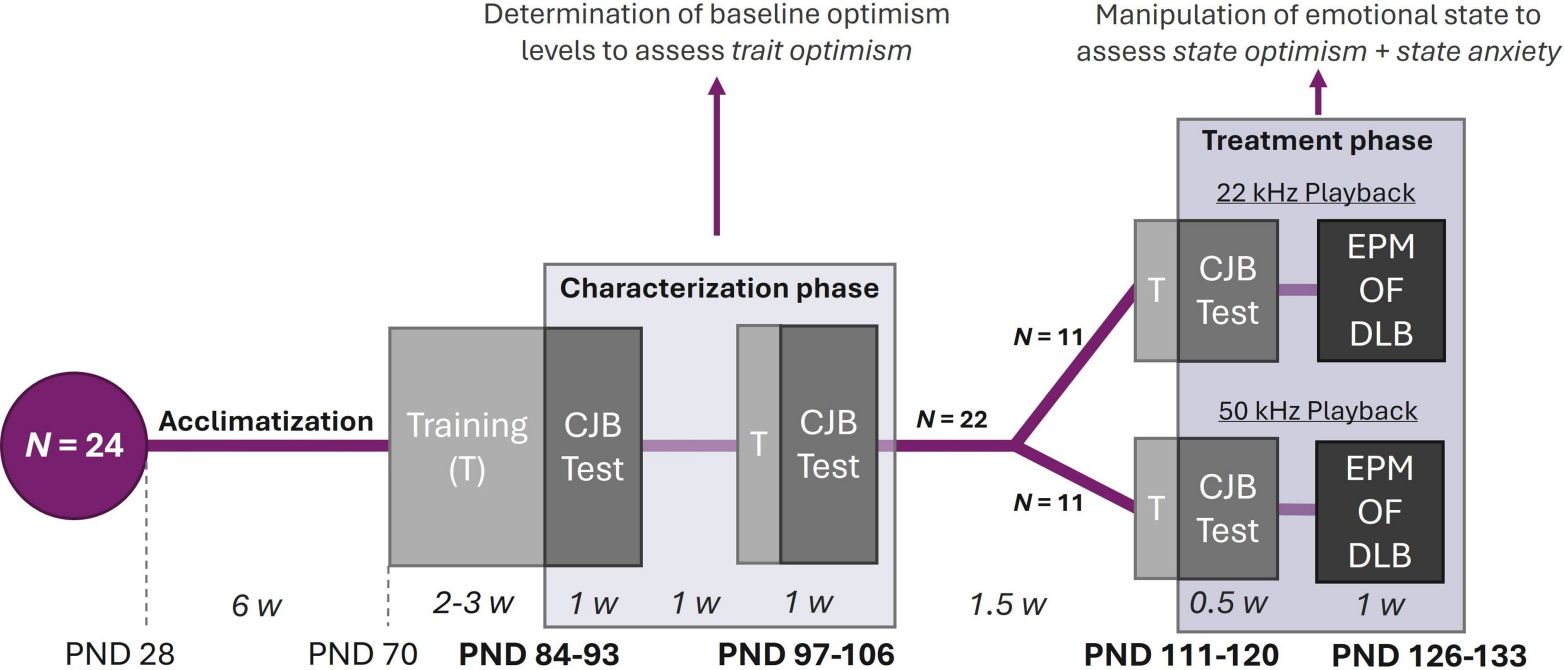
Experimental design. During the characterization phase, female rats were tested twice in the cognitive judgement bias (CJB) test for their optimism levels to assess trait optimism. Then, they were subjected to USV playback experiences in the treatment phase where they were tested again in the cognitive judgement bias test to investigate state optimism. Moreover, they were subjected to a battery of standard behavioral tests to assess additional indicators for a manipulation of emotional state: the elevated plus maze (EPM), open field (OF) and dark–light box (DLB) test. Duration of training, tests and intervals are shown in weeks (w), the age of the individuals is shown as postnatal days (PND).

More precisely, following delivery at postnatal day (PND) 28, animals were allowed to acclimate to the new housing environment for 6 weeks. During the last week of acclimatization, the rats were weighed daily to determine their bodyweights. At PND 70, the rats entered the experimental phase starting with training and the associated food restriction. The training was considered complete once all individuals of a batch simultaneously met a predefined learning criterion (see “Training” for details). The initial cognitive judgement bias training lasted 2–3 weeks, with one batch completing the criterion in two and the others in 3 weeks. Then, the characterization phase started at PND 84–93 with the first cognitive judgement bias test lasting for 3 days. After an interval of 1 week, the rats underwent a two-day reinforcement training (see “Training” for details) before being tested in the second cognitive judgement bias test. The characterization phase ended at PND 97–106. Optimism levels from the second cognitive judgement bias test were used to evenly assign rats to the two different USV playback treatments (each *N* = 11; see “USV experiences” for details). After 1 week, reinforcement training started again and was conducted for 2 days. Subsequently, the treatment phase began at PND 111–120. At this point, the rats were subjected to the USV playbacks in the third cognitive judgement bias test (see “USV experiences” for details). In the following week, the USV experiences were also applied during three standard behavioral assays: the elevated plus maze (EPM), the open field (OF), and the dark/light box (DLB) test. Each test was conducted once per subject, with a one-day interval between tests. The treatment phase was then concluded at PND 126–133 (see Figure 1).

All experimental procedures were conducted by the same experimenter (RM). The allocation of 22 or 50 kHz vocalization playbacks to individuals for the treatment phase, however, was performed by another person (LB) to blind the experimenter during cognitive judgement bias testing and the subsequent standard behavioral test battery.

### Cognitive judgement bias test

To characterize the individuals’ optimism levels, we conducted a cognitive judgement bias test originally developed by Burman and colleagues (54) and adapted by Richter and colleagues (55) to which we added more modifications (see “Paradigm”). The test is based on a spatial discrimination task to analyze responses toward an ambiguous location.

#### Apparatus

**Figure 2:**
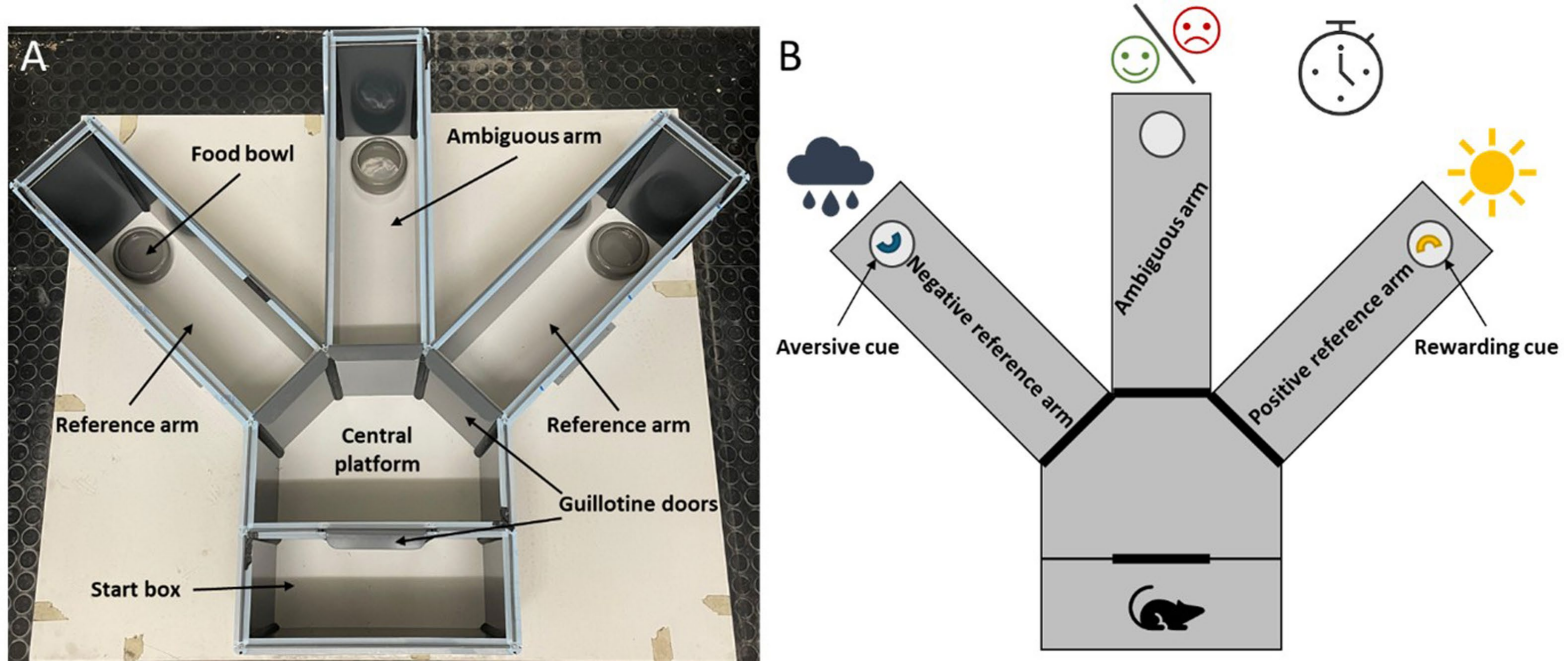
Cognitive judgement bias test apparatus and paradigm. **(A)** The apparatus comprised a start box, a central platform and three arms, all connected via guillotine doors. At the end of each arm, a food bowl was placed. **(B)** In the cognitive judgement bias test, rats were subjected to a three-arm maze for a spatial discrimination task. They learned to associate reaching the goal pot of a left or right arm with an unpalatable treat and reaching the goal pot of an opposite arm with a palatable reward. Then, they were presented with an intermediate arm and the latency to reach the goal pot was recorded. Slow responses were regarded as pessimistic, fast responses as optimistic.

The test apparatus consisted of a three-arm maze constructed from dark grey polyvinyl chloride (PVC). The entire apparatus had walls (height: 30 cm) and transparent lids to prevent the rats from escaping. It comprised a start box (floor space: 34 cm × 13.8 cm), three equally long arms (floor space: 49.5 cm × 13.8 cm) arranged at 45° angles, and a polygonal central platform (floor space: 23.9 cm × 34 cm) connecting the start box to the arms. Guillotine doors (35 cm × 13.1 cm) allowed manual separation of the start box and each arm from the central platform. A food bowl (diameter: 10 cm) was positioned at the distal end of each arm, centered approximately 2 cm from both side walls and the rear wall. The apparatus was wiped with 70% ethanol before the first and between sessions of different individuals to exclude any olfactory cues from conspecifics (Figure 2).

#### Procedure

For each cognitive judgement bias training and test round, the rats participated in one session per day with a break of maximally 2 days in between. The rats were trained and tested in a room adjacent to the housing room. Training and testing sessions were conducted exclusively in the dark phase between 9.15 a.m. and 5.15 p.m. under red light. The order of individuals trained or tested was randomized to account for daytime effects. Each session ended either when the rat completed all trials or after reaching a cut-off point of 40 min. If the session was not completed within this time, it was repeated on the following day for the respective animal. At the end of the session, the rat was carried back to its home cage. When all animals finished their training or test session, they received the estimated amount of food pellets according to their current weight.

#### Paradigm

Based on the previously established cognitive judgement bias paradigms (55, 56), the rats first needed to discriminate between a rewarded and a non-rewarded location, namely the left or the right arm of the maze. At the end of the positive arm, they were presented with half a palatable honey loop while in the negative condition, the honey loop was soaked in 2% hemi-sulfate quinine solution, making it unpalatable for the rats. Which arm was associated with the positive and negative condition was balanced across all individuals. The latency of the rats to dip their nose in the goal pot at the end of each arm was recorded. The rats proceeded to the test as soon as they reached a certain learning criterion (see “Training II). In the test, they were presented with the middle arm as an ambiguous location. The shorter the latency, the more optimistic an individual was considered.

Compared to the test design of Richter and colleagues (55), we made several modifications. Firstly, instead of five different locations with three ambiguous arms, we only used one ambiguous middle arm. Since the ambiguous trials have to be interspersed within the positive and negative reference trials, the number of ambiguous trials which can be presented per session is limited. Using only the ambiguous middle arm, we intended to gain more datapoints for this single measure of optimism levels by showing the middle arm more often than possible with three ambiguous cues. This practice has generally been applied in different cognitive judgement bias test designs [e.g., (57, 58)]. For spatial judgement bias tests like the one used in our study specifically, it has been shown that the middle arm provokes the highest level of ambiguity when mice (59) and rats (60) were presented with three ambiguous locations, supporting the use of only one arm. Secondly, we shortened and slightly modified the habituation to the apparatus. Thirdly, we performed one more training step (see “Training II”) for extended partial reinforcement to prepare the rats for the outcome of ambiguous trials. Fourthly, we reduced the reward size to avoid overfeeding of the rats after multiple trials per session. Fifthly, we only used one latency measure—the latency to reach the goal pot—instead of two since there was no measurable time difference to touching the reward. Sixthly, besides using the absolute latency in the ambiguous arm as a measure of optimism level [cf. (54, 55)], we also calculated relative latencies (see “Statistical analysis”). They set the latency to reach the goal pot in the ambiguous arm in relation to the latencies in the reference arms to control for running speed. Seventhly, we pseudorandomized the order of trials instead of a fixed schedule for positive, negative and ambiguous trials.

#### Training

The rats needed to participate in several training steps to learn the spatial discrimination task before they could be tested for their optimism levels in the cognitive judgement bias test. In more detail, they were required to associate one reference arm with a positive, rewarded condition and the other reference arm with a negative, unrewarded condition. First, the animals were habituated to the whole apparatus and rewards for two consecutive days (Habituation). Then, the training commenced with only one of the two reference arms open and the respective reward or unpalatable honey loop presented (Training I). Lastly, partial reinforcement was extended to prepare the rats for unrewarded ambiguous trials in the following test (Training II).

##### Habituation

During two consecutive days, the rats were habituated to the apparatus for 10 minutes per day. More specifically, the individual was placed in the start box of the apparatus with the door to the central part open but doors to the three arms closed. To accustom the rats to the rewards, 10 halves of honey loops were scattered on the floor of the start box and the central area. The rats were allowed to explore the two areas and consume these rewards for 4 minutes. Then, the doors to the three arms were opened as well, and the rats were given another 6 minutes to explore the whole apparatus. Then, the rat was taken out of the apparatus and returned to its home cage.

##### Training I

In the next step, the rats proceeded to the discrimination training. During training sessions, only one of the two reference arms was open. The rat was placed in the start box with the door closed for a 1-min acclimatization before the first training trial started. In the positive condition, the rats received one half of a honey loop and in the negative condition, half a honey loop soaked in quinine solution was presented at the end of the arm. The latency of the rat to dip its nose in the goal pot was recorded and the rat was allowed to consume the reward before being placed back in the start box. If the rat did not reach the goal pot within 120 s, the trial was stopped and the next trial started. Training I comprised 12 trials: the negative and the positive arm were presented six times, respectively. One of the positive trials was not rewarded to accustom the rats to non-rewarded ambiguous trials in the test. The sequence of trials was pseudorandomized following a number of rules. Firstly, the same arm could be shown only two times in a row. Secondly, the number of positive and negative arm presentations was balanced over the first and second half of each session (i.e., 3 presentations of negative and positive trials per half, respectively). Thirdly, the first two trials of a session were always one positive and one negative trial. Fourthly, the non-rewarded positive trial was conducted in the second half of the session. To proceed to the next training step, the rats were required to meet a learning criterion. More specifically, the latency to reach the goal pot in the negative and positive arm needed to differ significantly (Mann–Whitney *U* Test, one-tailed, *p* ≤ 0.05). However, since the test (see “Test”) followed directly after Training II and all rats were supposed to be tested simultaneously, the rats remained in Training I until all individuals of a batch met the criterion and the time schedule allowed training and testing on four consecutive days.

##### Training II

Similar to the previous training step, Training II began with a 1-min acclimatization phase in the start chamber followed by positive and negative trials with one reference arm open. The same rules for the trial sequence as in Training I applied, however, this training step included 14 trials in total: one more negative trial and one more unrewarded positive trial to extend the partial reinforcement for the subsequent test sessions. As in Training I, the latency to reach the goal pot needed to differ significantly between the negative and the positive reference arm (Mann–Whitney *U* Test, one-tailed, *p* ≤ 0.05).

##### Reinforcement training

Prior to the second and third cognitive judgement bias test, a short reinforcement training was conducted. It was limited to 2 days of training for all rats, with 1 day of Training I and another day of Training II.

#### Test

The first test day was always the same for all individuals of a batch. Testing was conducted on three consecutive days. The test sessions comprised 15 trials: In addition to six positive and six negative trials, each session included three ambiguous trials. While all positive trials were rewarded, ambiguous trials were unrewarded to avoid reinforcing optimistic or pessimistic responses. The rats were accustomed for unrewarded trials in the previous training steps which is a common practice in cognitive judgement bias studies (21, 57, 61). The trial sequence was modified according to the following criteria. Firstly, at least one positive and one negative trial were conducted before the first ambiguous trial and between subsequent ambiguous trials. Secondly, sessions did not conclude with an ambiguous trial. Thirdly, the trials preceding ambiguous trials were balanced over the three test days for positive and negative arms.

### USV experiences

During the treatment phase, USV playbacks of conspecifics were used to induce shifts in the emotional state of the rats. Two different frequencies were used: 50 kHz and 22 kHz. These frequencies were previously shown to influence cognitive judgement bias (26) as well as anxiety-like behavior (62).

#### Allocation of individuals to USV treatments

After the characterization phase, the rats were distributed to either the 50 kHz or 22 kHz treatment group by balancing individuals of differing optimism levels between the two treatments. More specifically, we used the individuals’ relative latencies (see “Statistical Analysis”), which were calculated using the mean latencies in the reference arms and ambiguous arm of the second cognitive judgement bias test. For the distribution to the two treatment groups, results of the second cognitive judgement bias test were used as the most recent indicators of optimism levels. The individuals of a batch were ranked based on the relative latencies as optimism levels. The “most optimistic” individual was then randomly assigned to one of the two treatment groups, the “second most optimistic” to the other treatment group and so on. This way, individuals of differing optimism levels were present in both treatment groups. Two animals were excluded due to health problems unrelated to the experiment which became apparent during the second cognitive judgement bias test before treatment allocation. The excluded animals belonged to two different batches, reducing these batches from eight to seven animals each. To maintain balanced treatment group sizes across the full sample, treatment allocation in the final batch was adjusted so that four animals were assigned to the group with the smaller sample size. Note here, however, that we chose to prioritize balancing out different optimism levels between the treatments over balancing out individuals from different cages since the interplay between trait and state optimism was our main point of interest.

#### Origin and processing of USV recordings

**Figure 3:**
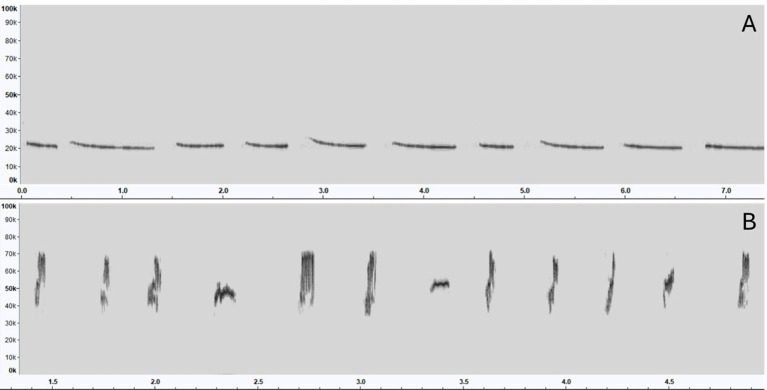
Spectrograms of the 22 kHz **(A)** and the 50 kHz vocalizations **(B)**.

The USV recordings were supplied by another research group who used them to study emotional contagion in rats (26). They recorded vocalizations from four male Sprague–Dawley rats during exploration of a sound-attenuated chamber. The recordings were made using an UltraSoundGate system and Recorder-USG software (Avisoft Bioacoustics e. K., Glienicke, Germany), and afterwards processed using Avisoft-SASLab Pro (Avisoft Bioacoustics e. K., Glienicke, Germany), applying a bandwidth filter for classification and noise reduction. Final output consisted of individual waveform files containing either 50 kHz or 22 kHz vocalizations, each approximately 7 s in duration and comprising multiple syllables in their original emission sequence [for further details, see original study by Saito and colleagues (26); Figure 3].

#### Composition of USV playback segments

For the purpose of this study, the 7-s-long audio files were presented in a randomized order, with a 13-s interval between each playback. Playback order was randomized using the built-in playlist randomization function of the SASLab Pro software (Avisoft Bioacoustics e. K., Glienicke, Germany). The rats were exposed to a total of 30 caLL segments (i.e., audio file and 13-s interval) during the 10-min-long treatment in cognitive bias test sessions (see “Procedure” for more details) and to 15 caLL segments during each of the three 5-min-long behavioral tests. These call segments were composed of different audio files, depending on the call type (i.e., 22 or 50 kHz). For the 50 kHz treatment, three different 7-s-long audio files were selected. Because of the different playback durations, each of these files was included 10 times in the cognitive bias test playback compilation and five times in the standard behavioral test playback list. For the 22 kHz treatment, six of the 7-s-long audio files were chosen and five copies of each were incorporated into the cognitive bias test playlist. For the standard behavioral tests, five of the six files were chosen, and three copies of each file were used. The identifiers of the files for the different playlists can be found in Supplementary Table S11.

#### USV playback equipment

Applying the two different treatments, playback of the recorded USVs was conducted using SASLab Pro software (Avisoft Bioacoustics e. K., Glienicke, Germany) via the Avisoft UltrasoundGate Player 116H and an Ultrasonic Dynamic Speaker Vifa (Avisoft Bioacoustics e. K., Glienicke, Germany). A recording system comprising an Ultrasound Microphone CM16/CMPA and UltraSoundGate 116 Hb (Avisoft, Glienicke, Germany) was employed to verify audio volume levels at approximately 60 dB within the respective apparatus prior to each standard behavioral test and to monitor sound output during the cognitive judgement bias test. Note here that we did not measure the sound intensity at multiple locations in the cognitive judgement bias test apparatus, however, the space was rather limited (27.7 cm × 34 cm). During the tests for anxiety-like behavior, the speaker was mounted centrally above the respective apparatus to minimize differences in sound intensity between areas. Although we cannot exclude low variation in sound intensity, we do not expect marked differences depending on the position of the rat in the area. Spectrograms were checked for background ultrasonic noise but visual inspection did not reveal any detectable ultrasonic noise.

#### USV playback procedure during the cognitive judgement bias test

The USV playbacks were presented to the rats in the cognitive judgement bias test apparatus for 10 min before the start of the test. In more detail, the rats were allowed to move freely in the start chamber as well as the central area of the apparatus while the USV recordings were played to them. The speaker was mounted on a tripod and positioned in front of the start box in a distance of approximately 15 cm, angled diagonally downward toward the apparatus. The microphone was placed on top of the middle arm, oriented toward the central area. Following the 10-min playback period, the one-minute interval preceding the first CJBT trial was used to remove the playback and recording equipment from the immediate vicinity of the apparatus. Since emotional states are transient and dependent on situational factors, we wanted to ensure that there was no time gap and change of place between treatment and test. At the same time, the playback should not directly interfere with the responses of the rats during cognitive judgement bias testing, for instance, through the location of the speaker.

#### USV playback procedure during behavioral tests

After a break of 1 week, the rats were subjected to a battery of standard behavioral tests to investigate the effects of the different USV playbacks on anxiety-like behavior. In contrast to the cognitive bias test, the USV playbacks were presented for the test duration of 5 min in the elevated plus maze, open field and dark–light box. Again, we considered it important to avoid any temporal or spatial separation between the positive and negative USV experiences and the behavioral tests. However, in contrast to the cognitive judgement bias test, the experiences were presented simultaneously with the measurement of anxiety-like behavior. This was necessary since testing of anxiety-like behavior should start immediately when the test arena is still novel for the animal (see “Behavioral testing”). Prolonging the time the rat spends in the apparatus could habituate the animal to the arena and confound measurements of anxiety-like behavior. The speaker was fixated above the respective apparatus, adjacent to a camera used for tracking animal behavior (see “Behavioral testing”).

### Behavioral testing

After the third cognitive judgement bias test, the rats were subjected to a battery of three standard behavioral tests, the elevated plus maze, the open field and the dark–light box test, widely applied to measure “state anxiety” (48, 50, 63). State anxiety is defined as the anxiety an individual experiences at a particular moment in time when facing a threatening stimulus (63, 64). More specifically, the tests are based on the rodents’ natural aversion toward brightly illuminated open spaces (65–67).

#### Procedure

The standard behavioral tests were conducted during the dark phase after 9.15 a.m. when the rats were active. The rats were tested in a different room than both their housing room and the room used for cognitive judgement bias training and testing. The order of individuals tested was randomized for each of three tests. Before the first test and between individuals, the respective apparatus was cleaned using 70% ethanol and paper towels. The rat was transported in a semitransparent red plastic box (floor space: 22 cm × 22 cm; height: 15 cm) which was cleaned between individuals as explained before. The behavior of the rats in the three tests was recorded using a camera (Logitech HD Pro C920 Full HD-Webcam 1,920 × 1,080 Pixel, Logitech, Apples, Switzerland) and analyzed by a tracking software (ANY-maze Video Tracking Software, version 6.32, Stoelting, Wood Dale, United States). Each of the three tests lasted 5 min for which the experimenter left the room to exclude a bias. Afterwards, the animal was carried back to its home cage using the transport box.

#### Elevated plus maze

The elevated plus maze is a widely used behavioral test for rodents, relying on their tendency to avoid open and brightly lit areas (65, 66). It is commonly applied to evaluate anxiety-like behavior and exploratory locomotion (68, 69). The elevated plus maze apparatus had a plus-shaped design and was constructed from grey plastic. It consists of two opposing closed arms (floor space: 52 cm × 10 cm) and two opposing open arms of the same dimensions, connected by a central square platform (floor space: 10 cm × 10 cm). The closed arms were enclosed by 30 cm high walls, topped with transparent, flexible plastic barriers to prevent rats from climbing or jumping out. The open arms had a low 0.4 cm raised edge to provide grip for the rats as they explored near the edges. The entire structure was elevated 60 cm above the ground and positioned in a fixed orientation within the test room which remained consistent throughout all testing sessions. Overhead lighting provided an illumination level of approximately 25 lux. Prior to each test, the animal remained inside the transport box for a 1-min acclimatization period to ensure that the state of arousal was similar for all individuals before the test (70). Upon the start of the test, the rat was placed in the center of the apparatus, facing the open arm. Parameters measured included the relative time spent on the open arms, the number of entries into open and closed arms and the total distance traveled [cf. (71)]. One animal had to be excluded due to technical problems while recording the test.

#### Open field

Similar to the elevated plus maze, the open field test is used to evaluate anxiety-like behavior and exploratory locomotion in rodents, tapping on their aversion to open and brightly lit environments (67, 69). The open field apparatus was square in shape (floor space: 104 cm × 104 cm) with 40 cm high walls and was constructed from grey plastic. Transparent, flexible plastic barriers were placed on top of the walls to prevent rats from climbing on them. The area within 26 cm of the walls was designated as the peripheral zone, while the central 52 cm × 52 cm area was defined as the center zone. The arena was illuminated from above with a light intensity of approximately 35 lux. Again, before each test, the rat stayed inside the transport box for a 1-min acclimatization period to ensure that all animals were in a similar state of arousal (70). When the test started, the rat was placed in the front left corner of the apparatus, facing the wall. The recorded parameters included the time spent in the center of the apparatus, the number of entries into the center, and the total distance traveled [cf. (71)].

#### Dark–light box

The dark–light box test is a commonly used method for assessing anxiety-like behavior in rodents (72). It typically involves a small, dark compartment connected to a larger, brightly lit area and takes advantage of the approach-avoidance conflict rodents face when exposed to unfamiliar environments (73). The dark–light box was constructed from black plastic and measured a floor space of 106 cm × 36 cm and walls which were 36 cm high. It consisted of a smaller, enclosed dark compartment (floor space: 36 cm × 36 cm) with a removable black lid, and a larger, open compartment (floor space: 70 cm × 36 cm) with black walls and a transparent floor but no lid. The two sections were connected by a 10 cm × 10 cm opening, which could be manually closed using a sliding guillotine door. Upon entering the test room, rats were placed directly into the dark compartment. The connecting door remained closed for a one-minute acclimation period again to ensure a similar arousal level for all rats (70) before being opened to initiate the test. Parameters for assessing anxiety-like behavior included the latency to first enter the open compartment, the time spent within it and the number of entries made.

### Statistical analysis

For this experiment, we planned to use 24 rats based on an *a priori* power analysis with a medium effect size (*f* = 0.3) and a statistical power of 80% (G*power, version 3.1.9.7). During the course of the experiment, however, two rats were euthanized due to unrelated medical reasons, reducing the sample size to *N* = 22. Data analysis was conducted in R [(74); version 4.4.0]. Mixed models were fitted using the “lme4” package [(75); version 1.1-35.3] and “lmerTest” [(76); version 3.1-3]. All plots were produced with the “ggplot2” package [(77); version 3.5.1].

#### Differences between locations during the characterization phase

First, we fitted a linear mixed model (LMM) to analyze if rats were able to discriminate between the three locations during the characterization phase, i.e., the tests without treatment. To do so, we estimated differences in absolute latencies to reach the goal pot in the two reference arms and the ambiguous middle arm. The response variable (“absolute latency”) was transformed using logarithmic transformation to improve the distribution of model residuals. The LMM included the main point of interest “location” (positive, negative and ambiguous) as a fixed effect. Note that the ambiguous condition was shown nine times (see “Test”) while the two reference conditions were shown 18 times per test repetition. Moreover, we added “batch” as well as “test repetition” (from 1 to 2) as fixed effects. As random effects, we included “ID” (for the individual), “age” and “day” (from 1 to 9, since there were three test days per test repetition). “Cage” was initially considered as an additional random effect but was not retained in the final models because the estimated cage-level variance was zero and its inclusion resulted in singular model fits. Model residuals were assessed using the DHARMa package [(78); version 0.4.6]. Pairwise comparisons of conditions were performed with the emmeans package [(79); version 1.10.2; adjust = tukey].

#### Trait optimism

To estimate if optimism is consistent across time and can thus be considered a trait, we analyzed the stability of optimism levels in the cognitive judgement bias tests without any influence of a treatment (test 1 + 2). The stability of a behavior can be assessed by determining their repeatability (*R*). The repeatability is defined as the proportion of total phenotypic variance that is attributable to between-individual differences for repeated measures of a behavior in a population (80). We used the “rptr” package [(81); version 0.9.22] to compute adjusted repeatabilities (*R*_adj_) which are estimated after accounting for confounding fixed effects (80).

To analyze repeatabilites, we used two different response variables as optimism levels, the absolute latency, i.e., the latency to reach the goal pot in the ambiguous condition, as well as the relative latency. The latter set the latency to reach the goal pot in the ambiguous condition into relation to the latency of the positive and negative condition. This is important, as the absolute latency to reach the goal pot in the ambiguous condition could be confounded by the running speed or general exploration tendencies of the individuals. Thus, the relative latency was calculated according to the following equation:


$$\text{Relative latency}=\frac{\text{Latency}(\text{ambiguous})-\text{Latency}(\text{positive})}{\text{Latency}(\text{negative})-\text{Latency}(\text{positive})}$$

Thereby, a long latency to reach the goal pot in the ambiguous condition might not necessarily reflect a pessimistic response if the rat also took a longer time to reach the goal pot in the positive and negative condition (i.e., the animal is an overall “slow runner”). To meet the assumptions for a normal error distribution, we log-transformed the absolute latency, and applied a Yeo-Johnson transformation to the relative latency, which we had confirmed in LMM with the same model structure prior. The Yeo-Johnson transformation is an extension of the Box-Cox transformation and can be applied to data including negative values and zeros (82). We then calculated the repeatability for each of the two outcome measures response, adding “test repetition” (i.e., the first and second cognitive judgement bias test) and “batch” as a fixed effects and “ID,” “age” as well as “day” as random effects. To assess the uncertainty of the repeatability estimates, we calculated confidence intervals by applying parametric bootstrapping (*N* = 1,000, confidence level = 95%). We used likelihood ratio tests to test for statistical significance.

#### State optimism

To measure state optimism, we analyzed the behavior of the rats in the third cognitive judgement bias test during the treatment phase under the influence of the positive or negative USV experiences. To do so, we investigated again both the absolute latencies as well as the relative latencies in the cognitive judgement bias test. The aim was then to assess the influence of optimism levels of the characterization phase (i.e., trait optimism) on optimism levels in the treatment phase (i.e., state optimism). Again, we log-transformed the absolute latency and applied a Yeo-Johnson transformation to the relative latencies. Then, we built an LMM for each of the outcome measures with the response variable “state optimism,” which was the absolute or relative latencies of the third cognitive judgement bias test. As fixed effects, we chose an interaction of “trait optimism” (i.e., the mean absolute latency per measurement or mean relative latency per day of the first and second cognitive judgement bias test) and “USV treatment” (i.e., 22 kHz and 50 kHz playback) since we were interested in the interplay of the USV playbacks and underlying trait optimism. Additionally, we used “batch” as a fixed effect as well. As a random effect, we added “ID” for repeated measurements of individual rats, therefore our model had the structure state optimism ~ trait optimism*USV treatment + Batch + (1|ID).

#### Behavioral testing

To assess differences in anxiety-like behavior while under the influence of the different USV playback treatments, we fitted linear as well as general linear models, depending on the data structure of the various outcome variables from the three behavioral tests. Linear models assuming a Gaussian data distribution were used for the distance traveled and the time spent in the center area of the open field arena, the relative time spent on open arms in the elevated plus maze test as well as the latency to first enter and the time spent in the open area of the dark–light box. Data for the time spent in the center zone of the open field were log-transformed and the latency to enter the open area of the dark–light box were square-root-transformed to better fit a normal distribution. Apart from that, count data such as entries to the center zone of the open field and the open area of the dark–light box were modelled as Poisson distributions. Entries to open and closed arms in the elevated plus maze were modelled as proportions. To keep the model structure consistent over all tests, we fitted all models with “USV treatment” only as a fixed effect.

### Ethical note

All experimental procedures complied with the regulations regarding animal experimentation within Germany (Animal Welfare Act) and the EU (European Communities Council DIRECTIVE 2010/63/EU). This study was approved by the corresponding local (Gesundheits- und Veterinäramt Bielefeld, Nordrhein-Westfalen) and federal authorities (Landesamt für Verbraucherschutz und Ernährung NRW „LAVE,” reference number 81-02.04.2022.A101).

Throughout the entire experiment and beyond, the welfare of the animals was closely monitored. Moreover, only non-invasive methods were employed, and all testing took place during the active phase of the animals. The rats were housed in spacious cages, structured with multiple levels and various enrichment items. After the experiment, the rats were either retained in our housing facility, rehomed, or transferred to a collaborating partner.

## Results

### Differences between locations during the characterization phase

**Figure 4:**
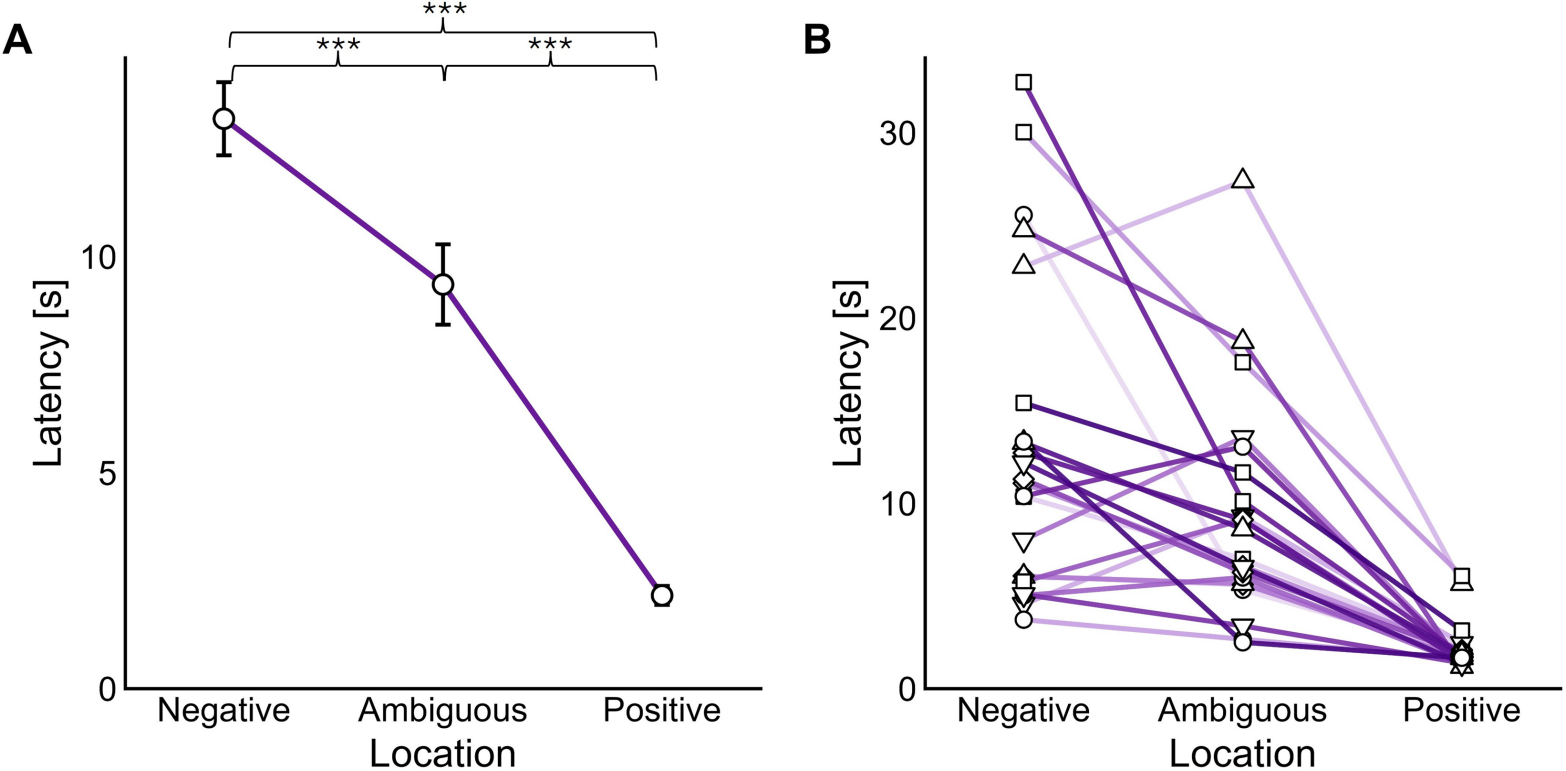
Differences in absolute latencies between the three locations during the characterization phase. **(A)** The latency of all rats (*N* = 22) to reach the goal pot at the end of the negative, ambiguous and positive arm is shown, pooled for all test repetitions. Dots show means, error bars indicate the standard error. Significance level: *** = *p* ≤ 0.001. **(B)** The individual latencies of the rats are shown, pooled for all test repetitions.

In a first step, we aimed to analyze if the animals could reliably differentiate between the three locations of the cognitive judgement bias test, i.e., the two reference arms and the ambiguous middle arm. We found a significant effect of “location” on latencies (*χ*^2^ = 965.0285, df = 2, *p* < 0.001; see Supplementary Tables S1, S2 for all effects). Subsequent pairwise comparisons revealed significant differences between all three locations (Figure 4; see Supplementary Table S3 for details), indicating that animals were able to discriminate between all three arms. Specifically, rats approached the positive location fastest and the negative location slowest, with latencies to reach the ambiguous location intermediate between these two reference locations.

### Trait optimism

**Figure 5:**
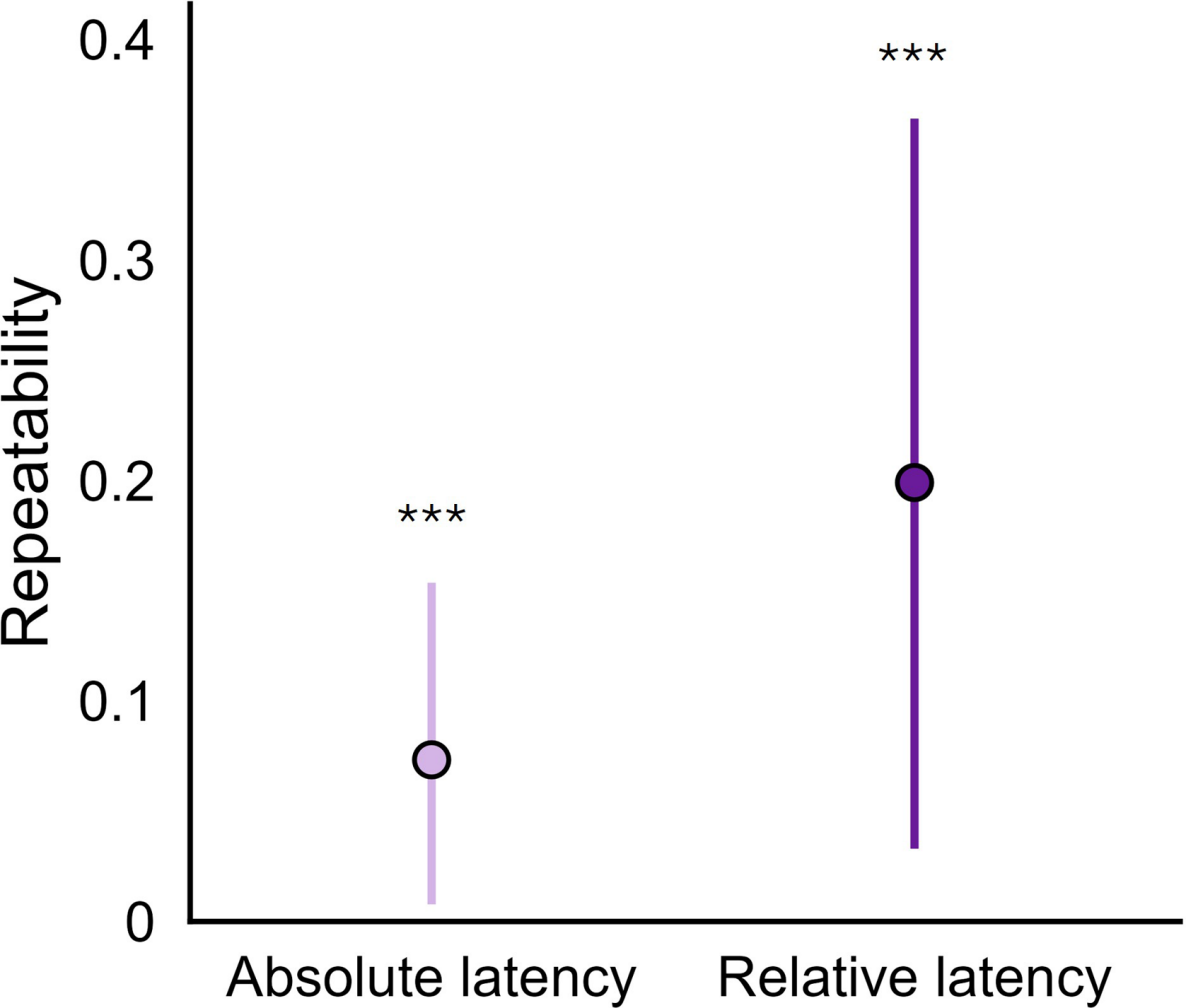
Adjusted repeatability estimates for optimism levels. The rats were tested twice in the cognitive judgement bias test to assess trait optimism. Optimism levels are shown as the absolute latency to reach the goal pot in the ambiguous arm and as relative latencies, which set the latency in the ambiguous arm in relation to the reference arms. Dots show repeatability estimates, lines represent the 95% confidence intervals. Significance level: *** = *p* ≤ 0.001.

In the next step, we estimated the stability of optimism levels during the characterization phase to assess trait optimism. Therefore, we calculated repeatabilities using both the absolute and the relative latencies to reach the goal pot in the ambiguous condition. We found optimism levels to be significantly repeatable when analyzing both absolute and relative latencies across the first two cognitive judgement bias tests, indicating that the rats behaved consistently. Interestingly, relative latencies were moderately repeatable (*R*_adj_ = 0.199, CI = [0.039, 0.366], *p* = <0.001) while the repeatability estimates for absolute latencies were lower (*R*_adj_ = 0.073, CI = [0.008, 0.154], *p* < 0.001; Figure 5). We also tested the repeatability of the absolute latencies in the positive and negative reference arms and found low but significant repeatability estimates for the rats’ behavior (see Supplementary Figure S1 and Table S4).

### State optimism

**Figure 6:**
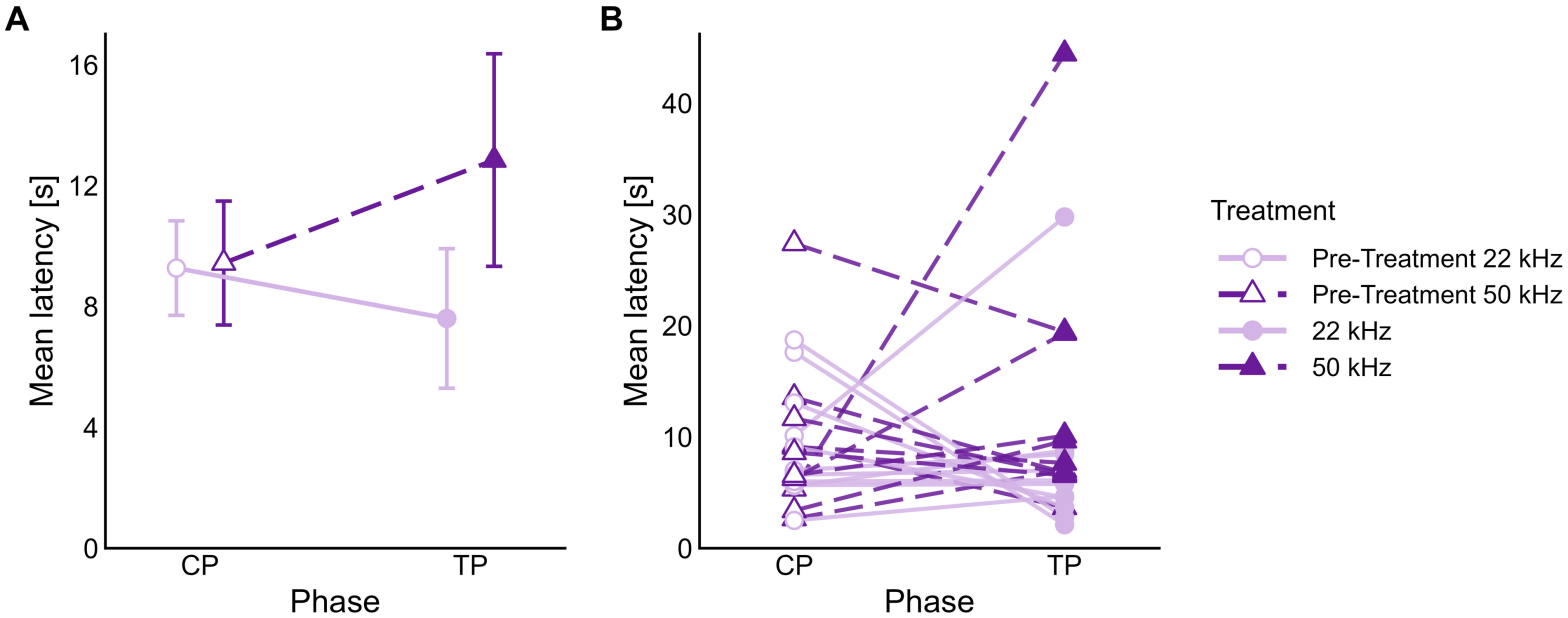
Effects of the USV treatment on absolute latencies in the ambiguous condition of the cognitive judgement bias test. **(A)** The mean latency to reach the goal pot in the ambiguous arm is shown during the characterization phase (CP; *N* = 22) as well as during the treatment phase (TP) after exposure to USV playbacks (*N*_50kHz_ = *N*_22kHz_ = 11). **(B)** Individual latencies to reach the goal pot in the ambiguous arm are shown before and after exposure to USV playbacks.

**Figure 7:**
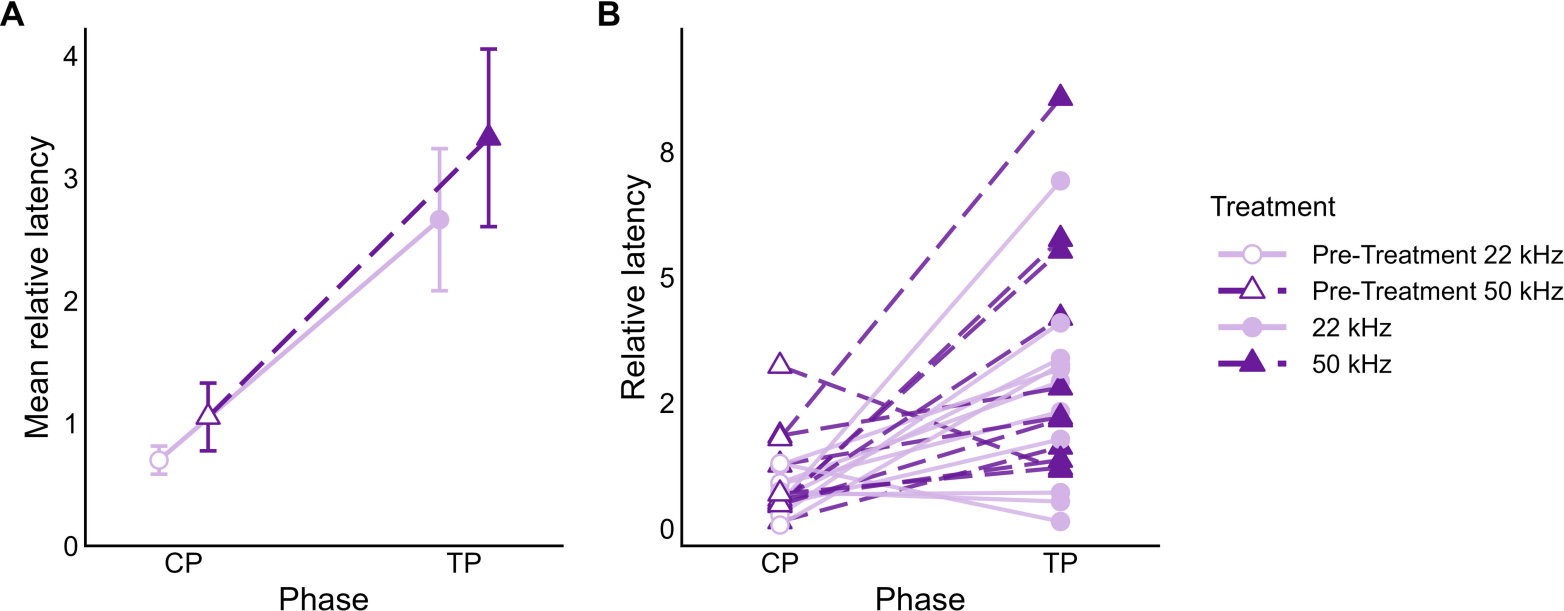
Effects of the USV treatment on relative latencies. **(A)** The mean relative latencies of the characterization phase (CP) as well as during the treatment phase (TP) after exposure to USV playbacks (*N*_50kHz_ = *N*_22kHz_ = 11) are shown. **(B)** Individual relative latencies are shown before and after exposure to USV playbacks.

Next, we fitted an interaction with the rats’ absolute and relative latencies during the first two tests (i.e., during the characterization phase) and the USV-treatment for the third test (i.e., during the USV treatment phase) to assess the influence of trait optimism and the USV playbacks on state optimism. For absolute latencies, trait optimism significantly affected optimism levels during the treatment phase (*χ*^2^ = 4.631, df = 1, *p* = 0.031), whereas no significant effect of trait optimism was found for relative latencies (*χ*^2^ = 0.124, df = 1, *p* = 0.725). This suggests that trait optimism may have influenced the emotional state of the animals during the treatment phase but this effect was not consistent across both outcome measures. Moreover, neither treatment nor an interaction of treatment and trait optimism had a significant effect, neither for absolute (Figure 6) nor relative latencies (Figure 7; Supplementary Tables S6–S9), indicating that the rat’s emotional state was not influenced by the USV playbacks.

### Behavioral testing

**Figure 8:**
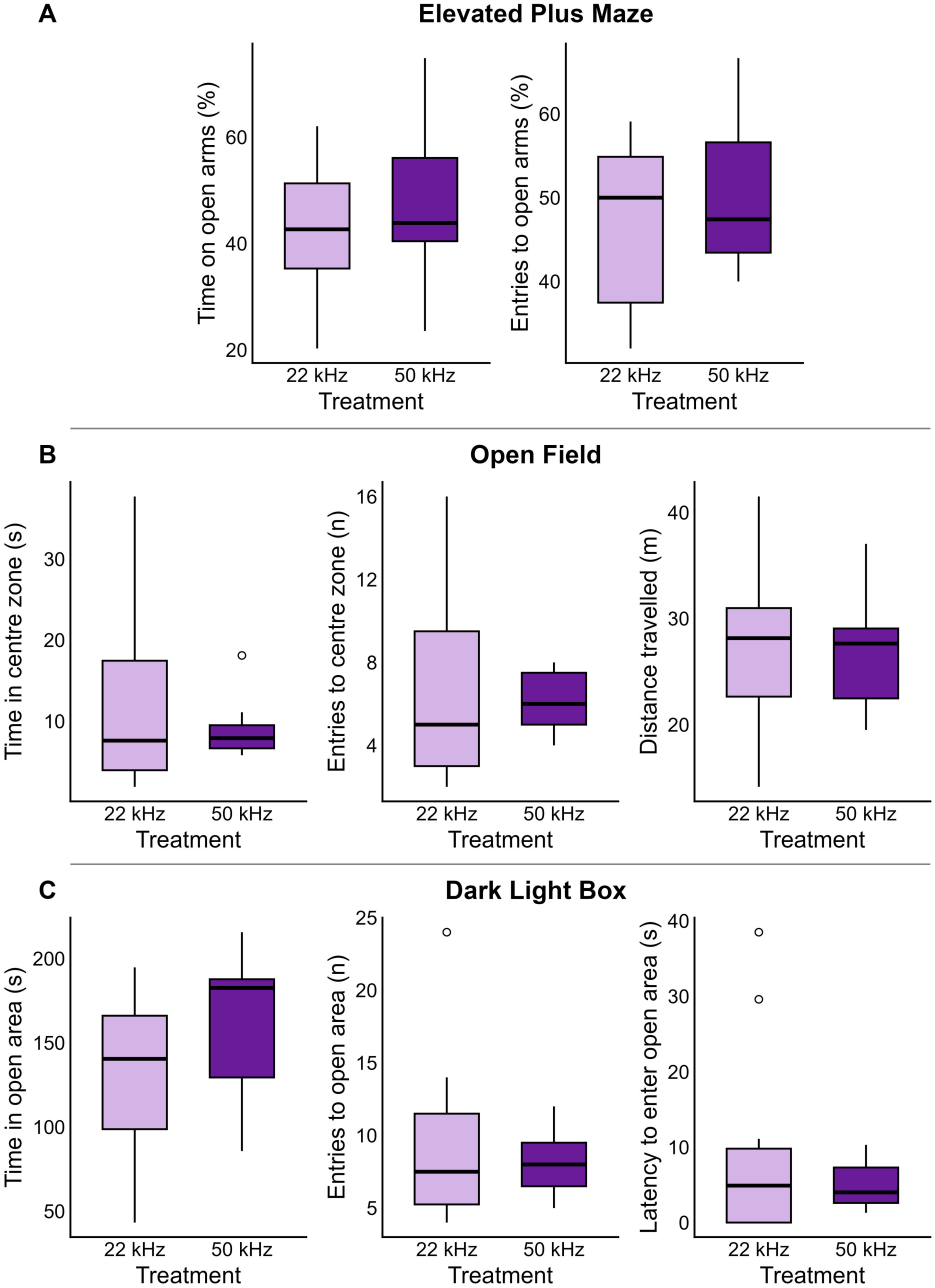
Behavioral tests. The rats were tested in a battery of tests to assess anxiety-like behavior under USV playback exposure. Displayed are parameters measured in the **(A)** Elevated plus maze **(B)** Open field and **(C)** Dark–Light box. Shown are the inter-quartile ranges (boxes) with medians (horizontal lines), 1.5*inter-quartile range (whiskers) and outliers (dots).

Finally, we assessed if the USV treatment induced differences in anxiety-like behavior in the elevated plus maze, open field and dark–light box test. Surprisingly, none of the parameters within the three behavioral tests revealed significant differences between the 22 kHz and 50 kHz groups, indicating that the USV playbacks did not differentially influence anxiety-like behavior (Figure 8; see Supplementary Table S10).

## Discussion

In the present study, we aimed to disentangle trait and state optimism using laboratory rats. Across the three-week characterization phase, we found significantly repeatable optimism levels, supporting the existence of a trait dimension of optimism. Notably, the repeatability estimates were higher for relative latencies (i.e., setting the latency at the ambiguous location in relation to the positive and negative reference locations) than for absolute latencies in the ambiguous arm, indicating that relative and absolute measures capture different aspects of the behavior in spatial judgement bias tests. Surprisingly, we did not find any statistically significant effects of 22- and 50-kHz USV playbacks on optimism levels or anxiety-like behavior during the following treatment phase, suggesting that neither state optimism nor any other behavioral measures were affected by playbacks. We discuss how future research might help to decompose trait and state dimensions of optimism and highlight implications for animal welfare, translational research and behavioral ecology.

### Consistent individual differences indicate a trait dimension of optimism

Across the two cognitive judgement bias tests conducted during characterization, optimism levels were significantly repeatable for both absolute and relative latencies, indicating that the rats responded consistently toward ambiguous cues. Therefore, our study provides further evidence for a trait dimension of optimism, aligning with previous findings for various species [e.g., (18–21, 83); but see (71)].

Interestingly, we previously did not find consistent individual differences in optimism levels within “early adulthood” in laboratory rats (i.e., PND 70-100), but only when calculating repeatability estimates across “early” and “full adulthood” [i.e., PND 86-175; (22)]. Adding to these findings, our present study indicates stabilization of optimism levels within a narrower time span between PND 84–106 that more or less overlaps in onset with the previously tested phase but ends substantially earlier. The suggested consolidation process, similar to the stabilization of personality traits in humans (84), may thus result in a detectable consistency of individual differences already earlier than previously assumed.

Overall, the estimates for both absolute and relative latencies were within the range of findings reported previously for the judgement of an ambiguous middle cue [(21): *R*_adj_ = 0.3; (22): from *R*_adj_ = 0.04 to *R*_adj_ = 0.09, depending on life phase]. However, comparing these two outcome measures among each other as well as to the reported average repeatability for behavioral parameters [*R* = 0.35; (85)], the repeatability estimate was rather low for absolute latencies (*R*_adj_ = 0.07) and moderately repeatable for relative latencies (*R*_adj_ = 0.2). Thus, optimism levels showed consistent individual differences, but a substantial proportion of variation remained unexplained. This variation may partly reflect age-related changes, learning or motivation across repeated testing, or, alternatively, may be induced by different stages of estrous cycle (22, 86).

Despite the widespread use of each of these measures in the literature [absolute latencies: (54, 55, 87, 88); relative latencies: (89–93)], the use of absolute latencies in spatial judgement bias tests has been discussed critically, since the outcome might be confounded by individual differences in exploration (94). However, while a higher exploratory drive might indeed lead to overall lower latencies in unknown or ambiguous situations, the here observed, rather low repeatability estimates do not hint toward a strong influence of a consistent exploration component. This interpretation is supported by the fact that exploration measures are known to be highly repeatable in rodents [reviewed in (95)], resulting in repeatability estimates that are way above the ones described here [e.g., (71): *R* = 0.5 to *R* = 0.9; (96): *R* = 0.7, (97): *R* = 0.7; (98): *R* = 0.7]. It is therefore likely that the observed latencies reflect more than exploration alone and instead may indeed capture a behavioral response to ambiguous cues.

### State optimism may be predicted by trait optimism but was not modulated by USV playbacks

Understanding the concept of optimism as representing a mixture of state and trait dimensions holds crucial consequences for various fields of research. For instance, while experimental stressors such as chronic restraint may have a negative effect on all tested animals, underlying trait pessimism was shown to intensify this effect (25), an important aspect to consider when assessing animal welfare. Moreover, trait pessimism may influence pharmacological responses. Drozd and colleagues (99) showed that trait pessimists were significantly less sensitive to negative feedback after antidepressant treatment than trait optimists, supporting the use of cognitive bias screening in translational contexts. Lastly, from an ecological perspective, being an optimist or pessimist can reflect alternative strategies with context-dependent fitness trade-offs. Thereby, in predictable environments, selection may favor consistent traits. In contrast, when conditions change rapidly, flexibility and state-dependent updating is necessary [e.g., (100–102)]. Therefore, a state–trait decomposition is central for linking judgement bias to adaptive decision-making.

To disentangle trait and state optimism, we therefore aimed to manipulate emotional state of trait optimists and pessimists by using positively valenced 50 kHz and negatively valenced 22 kHz USV playbacks. However, in contrast to our main hypothesis and previously reported findings of Saito and colleagues (26), exposure to 22 kHz and 50 kHz USV playbacks did not evoke any significant differences in optimism levels. Overall, these findings can be interpreted in two different ways: First, optimism levels may indeed be insensitive to state manipulations. Second, we were unable to detect treatment effects due to methodological limitations. With respect to the former, the literature reports various different findings.

Many studies on judgement biases in animals indeed detected changes in the emotional state as a consequence of experimental manipulations. For instance, Burman and colleagues (56) induced a negative cognitive bias by exposing rats to high light intensities during testing. Moreover, provision of environmental enrichment was often successfully used as a state manipulator [e.g., (54, 103)] and pharmacological treatments successfully modulated cognitive judgement bias as well [rats: (104); mice: (105)]. By contrast, other studies did not detect such effects after trying to modulate the animals’ emotional states. For example, a brief owner absence did not induce a negative judgement bias in pet dogs (106) and enriched vs. barren housing conditions did not alter cognitive judgement bias in pigs despite affecting other welfare-related measures such as cortisol levels (107). Together, these findings suggest that cognitive judgement bias is not uniformly sensitive to all putative state manipulations but might depend strongly on the type and strength of the treatment.

With respect to the latter, methodological limitations, such as habituation or the form of playback presentation (for more details, see next chapter), might have reduced the ability of USV playbacks to induce a reliable, directional shift in optimism levels in the present study. Notably, the study by Saito and colleagues (26), which successfully manipulated emotional state using USV playbacks, differed from our design in several aspects. They used male Sprague–Dawley rats and exposed them to the playbacks for 20 min prior to cognitive judgement bias testing. By contrast, we used female Lister Hooded rats and subjected them to the playbacks for only 10 min, as we observed signs of frustration when our rats were confined to the start chamber of the test apparatus during training. These differences may be relevant, as a recent playback study suggests that behavioral responses to 22 kHz and 50 kHz USVs can vary with sex and over the course of playback exposure (49). Moreover, estrous phase has been shown to influence judgement bias and anxiety-like behavior in female rats (86, 108), potentially contributing to within-individual variation in the present study. However, on the other side, a meta-analysis and review suggest that naturally cycling female rodents are generally not more variable than males when estrous stage is not controlled (109, 110), arguing against the assumption that estrous cycle was an important source of variation in the present study. Strain differences may also have contributed. For instance, the USV stimuli used in the present study were recorded from male Sprague–Dawley rats, whereas the receivers were female Lister Hooded rats. This sender–receiver mismatch may have reduced the ecological relevance of the playback stimuli, particularly because Lister Hooded and Sprague–Dawley rats differ in auditory processing (111). Moreover, the two strains have been reported to differ in anxiety-like behavior (112) and behavioral responses to aversive ultrasound have been shown to depend on strain (113). Thus, the effects of USV playbacks on optimism levels may depend on strain, sex, duration of playback exposure or the chosen cognitive judgement bias task. Instead of a bidirectional shift in optimism levels, methodological limitations may have caused the playbacks to act as a source of unspecific noise in the data, for example by increasing distraction or altering general arousal, rather than eliciting a clearly negative or positive state manipulation. Such introduced additional, non-specific variation by the USV playbacks may have also reduced between-individual consistency in decision-making. In line with this interpretation, we found repeatable optimism levels over the first two cognitive judgement bias tests, but the resulting “trait optimism” predicted optimism levels during the subsequent USV treatment phase only when absolute latencies were analyzed but not when relative latencies were used. This pattern suggests that consistent individual differences may have contributed to behavior during the treatment phase but that this effect was not robust across both measures. More specifically, absolute latencies may capture not only judgement bias, but also broader individual differences in exploration, motivation, or task engagement. In contrast, relative latencies control more directly for individual baseline differences in responses to the positive and negative reference cues. The interpretation of the USV playback effects may be further limited by the absence of additional behavioral recordings during playback exposure. Such recordings, for example of behavior in the start box, could have helped to assess whether the USV playbacks induced the intended specific responses, such as response vocalizations, freezing, or approach and avoidance behavior [cf. (38–40, 46, 47)]. However, this was not possible in the present study due to practical constraints. Additionally, some rats may have adjusted their behavior over the course of test repetitions as motivation declined after learning that ambiguous trials were not rewarded [cf. (10, 114, 115)]. Indeed, we found hints for an influence of repeated exposure to unrewarded ambiguous trials as the rats showed a tendency for increasing relative latencies over the characterization phase as well as significantly increasing absolute and relative latencies within the three test days of the treatment phase (see Supplementary Tables S12–S15 and Supplementary Figure S2). Thus, individual differences in learning about this outcome may have further weakened between-individual consistency.

Overall, experimental studies that aimed at disentangling state and trait dimensions of certain traits remain scarce. However, a potentially useful methodological approach comes from Varga and colleagues (116), who investigated the interaction between trait and state anxiety. They proposed that behavioral tests for anxiety-like behavior mainly reveal state anxiety, arising from interactions between an individual’s trait anxiety and the immediate environmental context. To better infer trait anxiety, they recommend refining both sampling and analysis by repeatedly assessing state anxiety across multiple test types in a semi-random order. Thereby, variation originating from individual trait and state dimensions, environmental context and their fluctuation over time can be captured. Following this approach, the decomposition of trait and state optimism could be refined. Thus, trait optimism would be determined by sampling state optimism either across multiple cognitive judgement bias tasks or repeated sessions in varying contexts to not only infer trait optimism from temporal but also contextual consistency. Despite the considerable practical and methodological challenges of implementing such an approach into already demanding judgement bias training and testing, it may also help to better account for undesired repeated-testing effects such as learning about unrewarded ambiguous trials or habituation to affect manipulations. Finally, summary measures would be derived to capture the individual’s average tendency across these state samples as trait optimism while within-individual deviations from this estimate would reflect state optimism, enabling a decomposition of the two dimensions.

### USV playbacks did not manipulate anxiety-like behavior

Besides the manipulation of cognitive judgement bias measures, our results also contradict previous research showing playback of 50 and 22 kHz USVs to modulate anxiety-like behavior in rats. Specifically, 22 kHz playback reduced open-area time and elicited an anxiety-consistent neural response in an elevated zero maze (117). Moreover, Bonauto and colleagues (49) showed 50 kHz playback to increase center exploration and locomotion, while 22 kHz USVs initially suppressed center exploration in an open field test. When given a shelter, 22 kHz playbacks decreased the time rats spent in an open area (50, 62), while 50 kHz playbacks led to increased exploration of the open area (50).

However, USV playback effects appear to be sensitive to experimental context and the behavioral readout used. For example, 22 kHz USV playback has been discussed as a translational tool for studying negative affective processing but its effects depend strongly on methodological and contextual factors (48). Similarly, responses to 50 kHz playback can vary between individuals and contexts, indicating that appetitive USV playback does not necessarily induce uniform behavioral responses across animals (118). Several factors may therefore explain why we did not detect clear playback effects in the present study: First, an important limitation of the present study is the absence of a non-USV control group. As we compared only 22-kHz and 50-kHz playbacks, it remains unclear whether one of the two stimuli failed to affect optimism, whether both failed to induce distinct valence-specific effects, or whether both produced a similar unspecific effect, for example through general arousal or habituation. Other studies suggest that USV treatment effects are not always most evident when different USV calls are compared directly. Instead, they may be better detectable relative to artificial tone controls (50, 62) or strongest in comparison to a control group of silence (117). Therefore, our findings should be interpreted against the background that USV playback effects are sometimes most apparent in specific contrasts (e.g., vs. silence or tone controls). Second, rats habituate rapidly in their responses to USV playbacks [cf. (119)]. For example, Schönfeld and colleagues (120) showed that male Long-Evans rats initially displayed robust approach behavior toward the speaker during 50 kHz playback and increased behavioral inhibition during 22 kHz playback, but approach behavior to 50 kHz declined markedly with repeated exposure across sessions. Our rats were exposed to the assigned USV playback for 10 min before each cognitive judgement bias test session. Because testing was conducted over 3 days, this resulted in 30 min of total playback exposure during the treatment phase, followed by additional exposure during the tests for anxiety-like behavior. Thus, habituation may have attenuated the effect of the USV playbacks on the rats’ behavior across the treatment phase. Third, treatment effects may be sensitive to the form of USV playback presentation. A study in male Sprague–Dawley rats showed that lower-intensity 22 kHz playback elicited more content-specific behavioral responses, whereas higher intensities produced more unspecific behavioral suppression that was less tied to the call category (121). As the playback volume used in our study was intermediate between these tested intensities, it may have fallen into a range in which responses were neither maximally content-specific nor strongly suppressive, thereby reducing the likelihood of detecting clear treatment effects. Lastly, from an ecological perspective, the lack of playback effects may also reflect a missing context. The 50 and 22 kHz playbacks were not linked to corresponding positive or negative outcomes such as reward and punishment, respectively. Especially with repeated exposure, they may have provided low predictive value in this setting. From the perspective of how animals use information to guide behavior, ignoring cues that do not help an animal to anticipate fitness-relevant consequences can be adaptive (122).

Taken together, the absence of treatment effects in our study may thus reflect a combination of sensitive parameter choices, rapid habituation under repeated exposure and low ecological relevance of non-contingent USV playbacks.

## Conclusion

The present study aimed to systematically investigate the link between trait and state optimism. From a broader perspective, disentangling trait and state optimism could be beneficial for refining translational research on pharmacological effects, improving animal welfare assessment and linking consistent vs. flexible decision-making to fitness consequences from an ecological perspective. Our findings contribute to the increasing evidence of optimism and pessimism to represent a consistent trait, as optimism levels were repeatable across two cognitive judgement bias tests separated by 3 weeks. However, the USV playbacks did not manipulate state optimism, highlighting the methodological challenges of inducing and detecting state-dependent shifts in cognitive judgement bias. Thus, whether trait optimism shapes responses to positive and negative experiences remains an open question, underlining the need for designs that enable the decomposition of consistent individual differences and context-dependent, within-individual variation in future studies. This might help to identify the mechanisms driving trait consolidation and rapid, state-dependent decision-making and to clarify the blurred boundary between the two dimensions.

## Data Availability

The datasets presented in this study can be found in online repositories. The names of the repository/repositories and accession number(s) can be found at: https://data.mendeley.com/datasets/8fy8ptvdxn/1.
