# Supplementary material for: Balancing consistency and flexibility: exploring trait and state optimism in rats using the cognitive judgement bias test
Source: Front Vet Sci. 2026 Jul 24;13:1849399. doi: 10.3389/fvets.2026.1849399 (PMC13447164; doi:10.3389/fvets.2026.1849399)
Supplement: Supplementary file 1 [file Data_Sheet_1.DOCX]

**Balancing Consistency and Flexibility: Exploring trait and state optimism in rats using the cognitive judgement bias test**

**Louisa Bierbaum^1^*, Rebecca Mainzer^1^, Carolin Mundinger^1^, Sylvia Kaiser^1,2^, S. Helene Richter^1,2^**

^1^Department of Behavioural Biology, University of Münster, Germany

^2^Joint Institute for Individualisation in a Changing Environment (JICE), University of Münster and Bielefeld University, Bielefeld, Germany

***Correspondence:** louisa.bierbaum@uni-muenster.de

# Supplementary material

**Table S1: Model summary of the LMM for the analysis of differences between locations – Absolute latency.**

|  | **Estimate** | **SE** | **z** | ***P*** |
| --- | --- | --- | --- | --- |
| (Intercept) | 1.781 | 0.238 | 7.496 | <0.001 |
| LocationNeg | 0.257 | 0.055 | 4.705 | <0.001 |
| LocationPos | -1.079 | 0.055 | -19.751 | <0.001 |
| Test_repetition2 | -0.128 | 0.218 | -0.589 | 0.598 |
| Batch2 | -0.305 | 0.274 | -1.111 | 0.304 |
| Batch3 | -0.225 | 0.261 | -0.859 | 0.424 |
| Batch4 | -0.094 | 0.261 | -0.359 | 0.732 |

**Table S2: ANOVA results for the analysis of differences between conditions – Absolute latency.**

|  | **χ^2^** | **df** | ***P*** |
| --- | --- | --- | --- |
| (Intercept) | 56.189 | 1 | <0.001 |
| Test_repetition | 0.347 | 1 | 0.556 |
| Location | 965.029 | 2 | <0.001 |
| Batch | 2.380 | 3 | 0.497 |

**Table S3: Pairwise comparisons of latencies between the three different locations.**

| **Comparison** | **Estimate** | **SE** | **z** | ***P*** |
| --- | --- | --- | --- | --- |
| N vs. M | -0.257 | 0.055 | -4.705 | <0.001 |
| P vs. M | 1.079 | 0.055 | 19.751 | <0.001 |
| N vs. P | 1.336 | 0.045 | 29.952 | <0.001 |

**Table S4: Repeatability estimates with confidence interval for the three different locations.** Likelihood ratio tests indicate significance (p).

| **Cue** | **R^2^** | **CI** | **p** |  |  |
| --- | --- | --- | --- | --- | --- |
| P | 0.052 | [0.014, 0.107] | <0.001 |  |  |
| M | 0.073 | [0.008, 0.154] | <0.001 |  |  |
| N | 0.122 | [0.046, 0.217] | <0.001 |  |  |
|  |  |  |  |  |  |

**Figure S1: Repeatabilities of latencies to reach the goal pot in the three conditions.** The rats were tested twice in the cognitive judgement bias test (N = 22). Dots show repeatability estimates, lines represent the 95 % confidence intervals. Significance level: *** = P ≤ 0.001.

**Table S5**: **Absolute variance of between- and within-individual differences.**

| **Location** | **Between-individual variance** | **Within-individual variance** |  |  |  |
| --- | --- | --- | --- | --- | --- |
| Negative | 0.154 | 1.071 |  |  |  |
| Ambiguous | 0.091 | 0.995 |  |  |  |
| Positive | 0.019 | 0.306 |  |  |  |

**Table S6: Model summary for analysis of state optimism – Absolute latency.**

|  | **Estimate** | **SE** | **z** | ***P*** |
| --- | --- | --- | --- | --- |
| (Intercept) | 1.057 | 0.310 | 3.411 | 0.001 |
| Treatment50kHZ | 0.493 | 0.371 | 1.328 | 0.187 |
| CBT1_2_log | 0.331 | 0.154 | 2.152 | 0.033 |
| Batch2 | -0.642 | 0.282 | -2.281 | 0.036 |
| Batch3 | -0.479 | 0.249 | -1.922 | 0.071 |
| Batch4 | -0.289 | 0.248 | -1.165 | 0.260 |
| Treatment50kHz:CBT1_2_log | -0.062 | 0.218 | -0.286 | 0.775 |
|  |  |  |  |  |

|  | **χ^2^** | **df** | ***P*** |
| --- | --- | --- | --- |
| (Intercept) | 11.635 | 1 | <0.001 |
| CBT1_2_log | 4.631 | 1 | 0.031 |
| Treatment | 1.764 | 1 | 0.184 |
| Batch | 6.085 | 3 | 0.108 |
| Treatment:CBT1_2_log | 0.082 | 1 | 0.775 |

**Table S7: ANOVA results for analysis of state optimism – Absolute latency.**

**Table S8: Model summary for analysis of state optimism – Relative latency.**

|  | **Estimate** | **SE** | **z** | ***P*** |
| --- | --- | --- | --- | --- |
| (Intercept) | 0.694 | 0.196 | 3.538 | 0.001 |
| Treatment50kHz | -0.066 | 0.235 | -0.282 | 0.779 |
| rel_mean_CBT1_2 | -0.120 | 0.340 | -0.352 | 0.726 |
| Batch2 | 0.122 | 0.170 | 0.714 | 0.486 |
| Batch3 | 0.043 | 0.152 | 0.284 | 0.780 |
| Batch4 | -0.090 | 0.149 | -0.601 | 0.556 |
| Treatment50kHz:rel_mean_CBT1_2 | 0.294 | 0.468 | 0.628 | 0.534 |

**Table S9: ANOVA results for analysis of state optimism – Relative latency.**

|  | **χ^2^** | **df** | ***P*** |
| --- | --- | --- | --- |
| (Intercept) | 12.518 | 1 | <0.001 |
| rel_mean_CBT1_2 | 0.123 | 1 | 0.725 |
| Treatment | 0.080 | 1 | 0.778 |
| Batch | 2.159 | 3 | 0.540 |
| Treatment:rel_mean_CBT1_2 | 0.394 | 1 | 0.530 |

**Table S10: Model summaries for all behavioural tests.**

| **Test** | **Parameter** | **Estimate** | **SE** | **z** | **P** |
| --- | --- | --- | --- | --- | --- |
| Elevated plus maze | Entries to open arms | 0.105 | 0.174 | 0.601 | 0.548 |
|  | Time on open arms | 5.565 | 6.055 | 0.919 | 0.370 |
|  |  |  |  |  |  |
| Open field | Entries to centre zone | -0.058 | 0.170 | -0.340 | 0.734 |
|  | Time in centre zone | -0.017 | 0.255 | -0.068 | 0.946 |
|  | Distance travelled | -0.600 | 2.753 | -0.218 | 0.830 |
|  |  |  |  |  |  |
| Dark light box | Latency to enter open area | -0.063 | 0.728 | -0.087 | 0.932 |
|  | Entries to open area | -0.150 | 0.148 | -1.014 | 0.311 |
|  | Time in open area | 32.57 | 20.62 | 1.579 | 0.131 |

**Table S11: Identifiers for files used in the USV playback lists.**

| **Treatment** | **Cognitive judgement bias test** | **Tests for anxiety-like behavior** |  |
| --- | --- | --- | --- |
| 50 kHz | 50-10.wav, 50-26.wav,  50-29.wav | 50-10.wav, 50-26.wav,  50-29.wav |  |
| 22 kHz | 22-1.wav, 22-6.wav,  22-10.wav, 22-15.wav,  22-18.wav, 22-21.wav | 22-6.wav, 22-10.wav,  22-15.wav, 22-18.wav,  22-21.wav |  |

**Table S12: ANOVA for analysis of learning about the outcome of ambiguous trials across the characterization phase – absolute latencies.**

|  | **χ^2^** | **df** | ***P*** |
| --- | --- | --- | --- |
| (Intercept) | 22.007 | 1 | <0.001 |
| Day | 8.728 | 5 | 0.120 |
| Batch | 1.983 | 3 | 0.576 |

|  | **χ^2^** | **df** | ***P*** |
| --- | --- | --- | --- |
| (Intercept) | 8.431 | 1 | 0.004 |
| Day | 10.646 | 5 | 0.059 |
| Batch | 2.641 | 3 | 0.450 |

**Table S13:** **ANOVA for analysis of learning about the outcome of ambiguous trials across the characterization phase – relative latencies.**

**Table S14: ANOVA for analysis of learning about the outcome of ambiguous trials within the treatment phase – absolute latencies.**

|  | **χ^2^** | **df** | ***P*** |
| --- | --- | --- | --- |
| (Intercept) | 3.893 | 1 | 0.048 |
| Treatment | 1.670 | 1 | 0.196 |
| CBT_1_2_Latency_log | 5.860 | 1 | 0.015 |
| Batch | 5.860 | 3 | 0.119 |
| Day_order | 9.827 | 2 | 0.007 |
| Treatment:CBT1_2_Latency_log | 0.057 | 1 | 0.811 |

**Table S15: ANOVA for analysis of learning about the outcome of ambiguous trials within the treatment phase – relative latencies.**

|  | **χ^2^** | **df** | ***P*** |
| --- | --- | --- | --- |
| (Intercept) | 6.685 | 1 | 0.010 |
| Treatment | 0.095 | 1 | 0.758 |
| CBT_1_2_Latency_log | 0.008 | 1 | 0.928 |
| Batch | 1.857 | 3 | 0.603 |
| Day_order | 9.180 | 2 | 0.010 |
| Treatment:CBT1_2_Latency_log | 0.427 | 1 | 0.513 |

**Figure S2: Absolute and relative latencies across all test days.**
